# COMPARING IN-PERSON ONLY, TELEMEDICINE ONLY, AND HYBRID HEALTH CARE VISITS AMONG OLDER ADULTS IN SAFETY-NET CLINICS

**DOI:** 10.1093/geroni/igad104.0476

**Published:** 2023-12-21

**Authors:** Omolola Adepoju, Patrick Dang, Maya Singh, Arlette Chavez

**Affiliations:** University of Houston, Houston, Texas, United States; University of Houston, Houston, Texas, United States; University of Houston, Houston, Texas, United States; University of Houston College of Medicine, Houston, Texas, United States

## Abstract

**Introduction:**

Older adults face challenges in seeking healthcare. This study examined factors associated with in-person only versus telemedicine only versus hybrid healthcare visits among adults 65+ in safety-net clinics.

**Methods:**

Data was obtained from a large Texas-based Federally Qualified Health Center (FQHC) network. The dataset included 12,279 appointments for 3,914 unique older adults between March and November 2020. The outcome of interest was a 3-level indicator of telemedicine visits: in-person visits only, telemedicine visits only, and hybrid (In person + Telemedicine) visits during the study period. We used a multinomial logit model adjusting for patient-level characteristics to assess the strength of the relationships.

**Results:**

Compared to their white counterparts, Black and Hispanic older adults were significantly likely to have telemedicine only visits vs in-person only visits (Black RR: 0.59, 95% CI 0.41-0.86; Hispanic RR: 0.46, 95% CI 0.36-0.60). However, there were no significant racial and ethnic differences in hybrid utilization (Black RR: 0.91, 95% CI 0.67-1.23; Hispanic RR: 0.86, 95% CI 0.70-1.07).

**Discussion:**

Our findings suggest that hybrid opportunities may bridge racial and ethnic disparities in access to care. Clinics should consider building capacity for both in-person and telemedicine opportunities as complementary strategies.

